# Albumin/Mitotane Interaction Affects Drug Activity in Adrenocortical Carcinoma Cells: Smoke and Mirrors on Mitotane Effect with Possible Implications for Patients’ Management

**DOI:** 10.3390/ijms242316701

**Published:** 2023-11-24

**Authors:** Aurora Schiavon, Laura Saba, Gianluca Catucci, Jessica Petiti, Soraya Puglisi, Chiara Borin, Giuseppe Reimondo, Gianfranco Gilardi, Claudia Giachino, Massimo Terzolo, Marco Lo Iacono

**Affiliations:** 1 Department of Clinical and Biological Sciences, University of Turin, 10043 Turin, Italy; aurora.schiavon@edu.unito.it (A.S.); laura.saba@unito.it (L.S.); soraya.puglisi@unito.it (S.P.); chiara.borin@edu.unito.it (C.B.); giuseppe.reimondo@unito.it (G.R.); claudia.giachino@unito.it (C.G.); massimo.terzolo@unito.it (M.T.); 2 Department of Life Sciences and Systems Biology, University of Turin, 10123 Turin, Italy; gianluca.catucci@unito.it (G.C.); gianfranco.gilardi@unito.it (G.G.); 3 Division of Advanced Materials Metrology and Life Sciences, Istituto Nazionale di Ricerca Metrologica (INRiM), 10135 Turin, Italy; j.petiti@inrim.it

**Keywords:** adrenocortical carcinoma, mitotane, albumin, human serum, H295R cell line, drug resistance

## Abstract

Background: Mitotane is the only drug approved for the treatment of adrenocortical carcinoma (ACC). Although it has been used for many years, its mechanism of action remains elusive. H295R cells are, in ACC, an essential tool to evaluate drug mechanisms, although they often lead to conflicting results. Methods: Using different in vitro biomolecular technologies and biochemical/biophysical experiments, we evaluated how the presence of “confounding factors” in culture media and patient sera could reduce the pharmacological effect of mitotane and its metabolites. Results: We discovered that albumin, the most abundant protein in the blood, was able to bind mitotane. This interaction altered the effect of the drug by blocking its biological activity. This blocking effect was independent of the albumin source or methodology used and altered the assessment of drug sensitivity of the cell lines. Conclusions: In conclusion, we have for the first time demonstrated that albumin does not only act as an inert drug carrier when mitotane or its metabolites are present. Indeed, our experiments clearly indicated that both albumin and human serum were able to suppress the pharmacological effect of mitotane in vitro. These experiments could represent a first step towards the individualization of mitotane treatment in this rare tumor.

## 1. Introduction

Mitotane, or o,p′-DDD (1-chloro-2-[2,2-dichloro-1-{4-chlorophenyl}ethyl] benzene), is the only drug approved for the treatment of adrenocortical carcinoma (ACC), being commercially available as Lysodren^®^ (HRA Pharma Rare Diseases, Paris, France). Mitotane is a parent compound of the insecticide DDT (dichlorodiphenyltrichloroethane), from which it was isolated in 1940. In 1959, the efficacy of mitotane was reported for the first time in male patients with ACC [1], and in 1970 it was cleared by the Food and Drug Administration for the treatment of advanced ACC. Currently, mitotane remains the cornerstone of medical therapy for advanced ACC, either as a single drug or in combination with classical cytostatic agents [2]. Mitotane is also increasingly used in the postoperative adjuvant setting [3,4,5,6].

Mitotane exerts its pharmacological effects primarily on the adrenal cortex, in particular in the zona fasciculata and zona reticularis, leading to an impairment of steroidogenesis and cell destruction [7,8]. In the mitochondrial membrane, mitotane is converted to DDE (1,1-(o,p′-dichlorodiphenyl)-2,2 dichloroethylene) and DDA (1,1-(o,p′-dichlorodiphenyl) acetic acid) through α and β hydroxylation, respectively [9]. These transformations are mediated by the CYP450 family, an active enzyme system that is highly expressed in the adrenal gland. It has been demonstrated that DDE is an active metabolite of mitotane, although it is present at low plasma concentrations, while it is still debated whether DDA is active or not [10,11].

Despite 50 years of clinical use, the mechanism of action of mitotane remains unclear. Its anti-steroidogenic and antitumor effects seem to be mainly due to the inhibition of steroidogenesis and induction of endoplasmic reticulum (ER) stress resulting in cell death. It is not yet known whether mitotane inhibits key enzymes of steroidogenesis, steroidogenic regulatory genes, or both [9]. Studies showed that ER stress represents a key molecular pathway activated by mitotane through the inhibition of SOAT1 (Sterol-O-acyl transferase 1) [12]. The blocking of SOAT1 leads to free cholesterol and fatty acid accumulation and the induction of ER stress, promoting the downregulation of genes involved in lipid metabolism and steroidogenesis [12]. Mitotane-mediated apoptosis and/or necroptosis can also be induced by the inhibition of mitochondrial respiratory chain complexes I and IV and disassembly of mitochondria-associated membranes [13].

In vitro experiments are an essential tool to investigate the mechanism of action of any drug at the molecular level. For mitotane, the most-used cell models are H295-derived cell strains established from a female patient with ACC. Since the creation of the original H295 strain, many laboratories have tried to explore the cytotoxic ability of mitotane with mixed success. Indeed, studies have shown that the mitotane IC50 varied through a wide range of concentrations, from 40–60 μM up to 100–200 μM [13]. Intriguingly, Hescot et al. identified an opposite correlation between mitotane’s effect and lipoprotein concentration in culture media, indicating that mitotane exerted its toxic effects more efficiently when cells were grown in a lipoprotein-free medium [14]. The observation that mitotane’s action was strongly influenced by the culture conditions is also confirmed by other authors [15,16,17]. In an effort to reduce experimental variability, M. Kurlbaum et al. developed a practical guide recommending the verification of cell strains, consistent use of cell passages, uniform medium sourcing, and evaluation of hormone/steroid content in the culture medium [16].

This study aims to clarify the impact of confounding factors like albumin on the activity of mitotane and its metabolites.

## 2. Results

### 2.1. Study of the Influence of BSA and Serum on the Pharmacological Effect of Mitotane and Its Metabolites

**Figure 1:**
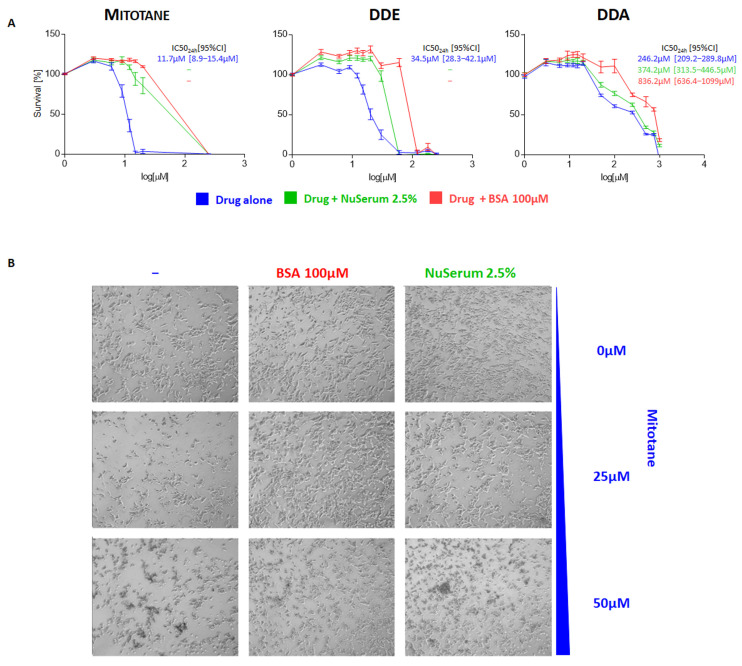
Cytotoxic and phenotypic effect of mitotane and its metabolites in H295R cell model. (**A**) Concentration–response curve showing drug-induced inhibition of cell viability of H295R cell model. H295R cells were cultured for 24 h testing different drug concentrations (mitotane and DDE 0–250 µM; DDA 0–1 mM). For each drug, the range of increasing concentrations was selected to maximize the sigmoidal pattern of the dose–response graph. For each drug, this standard condition was “perturbed” by adding a fixed amount of BSA (100 µM) or NuSerum (2.5%) to each curve concentration point. Technical quadruplicates were performed for each drug condition and the experiments were replicated at least 2 times. “−” indicates an unreliable evaluation of IC50_24_. (**B**) H295R cells were cultured for 24 h with different concentrations of mitotane alone or in the presence of BSA 100 μM and NuSerum 2.5%. BSA and NuSerum clearly counteracted the toxic effect of 25 μM mitotane on H295R, as observed in the first column. In fact, it is shown that the addition of the two additives preserves the physiological cellular morphology with respect to the experimental conditions with 25 μM mitotane alone. The protective effect, although present, is less evident at higher drug concentrations (50 μM).

A viability assay was performed to test the hypothesis that lipoproteins and serum globulins may affect the in vitro activity of mitotane [14,15,16,17]. Figure 1 shows the effect of mitotane, DDE, and DDA, respectively, on H295R cell viability. Mitotane had the highest effect on cells at 24 h with an IC50_24_ of 11.7 μM [95% confidence interval (95%CI): 8.9–15.4 µM], followed by DDE with an IC50_24_ of 34.5 μM [95%CI: 28.3–42.1 µM] and, lastly, DDA with an IC50_24_ of 246.2 μM [95%CI: 209.2–289.8 µM] (Figure 1A, blue line). By adding BSA 100 μM (Figure 1A, red line) or NuSerum 2.5% (Figure 1A, green line) to paired drug concentrations, mitotane and DDE efficacy was dramatically reduced, while DDA was less affected by the presence of these substances. A significant biological effect of mitotane and DDE was observed only with supersaturated drug concentrations, which did not permit a reliable evaluation of IC50_24_. Moreover, the DDA IC50_24_ of 246.2 μM was increased to 374.2 μM with NuSerum 2.5% [95%CI: 313.5–446.5 µM] and to 836.2 μM with BSA 100 μM [95%CI: 636.4–1099 µM]. These results were also confirmed by the morphological and structural changes in treated cells: BSA 100 μM and NuSerum 2.5% clearly contrasted the mitotane effect (Figure 1B, column one versus columns two and three).

**Figure 2:**
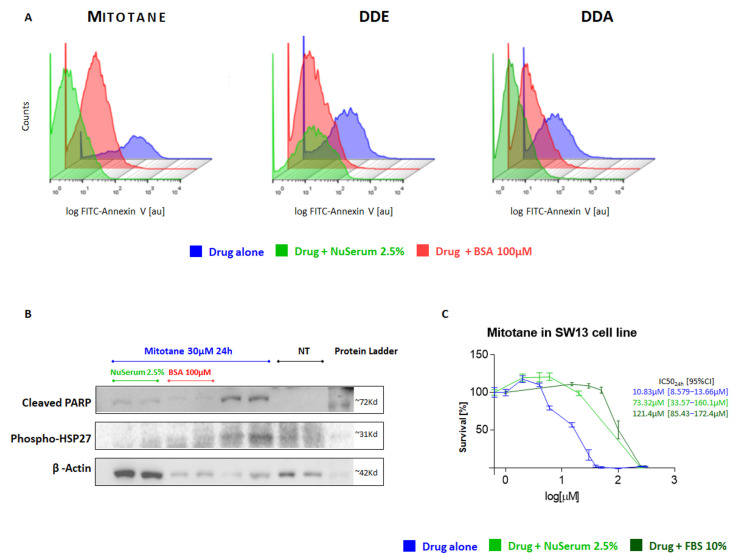
(**A**) Cytotoxic effect of mitotane and its metabolites in H295R cell model. Apoptosis analysis. H295R cells (1.5 × 10^5^ cells/well) were seeded in 24-well plates in complete medium. After 24 h incubation with the appropriate concentration of each drug in study and with or without additive substance, cells were detached and decorated with FITC-Annexin V antibody. FACS analysis was performed with cell sorter/FACS facility. (**B**) Western blot analysis for the detection of apoptosis-related proteins. H295R cells were cultured for 24 h with mitotane 30 μM alone (blue) and with the addition of BSA 100 μM (red) or NuSerum 2.5% (green). A 30 μg volume of protein lysate from each condition in biological replicate was evaluated for apoptosis-related proteins by decorating with cleaved-PARP (89 kDa) and phospho-HSP27 (27 kDa) antibodies. (**C**) Cytotoxic effect of mitotane in NCI-SW-13 cell model. Concentration–response curve of drug-induced inhibition of cell viability in SW-13 cell model. SW-13 cells were cultured for 24 h with a range of mitotane concentrations (0–250 µM). This standard condition was “perturbed” by adding NuSerum 2.5% or the physiological condition (FBS 10%). Technical quadruplicates were performed for each drug condition and the experiments were replicated at least 2 times.

Similar results were also observed by evaluating cellular apoptosis induced by mitotane and its metabolites with FACS analysis. For all drugs in this study, we found significant lower apoptosis with the addition of BSA 100 μM (Figure 2A, red area) or NuSerum 2.5% (Figure 2A, green area) compared to using the drugs alone (Figure 2A, blue area). To confirm the mitotane-mediated apoptosis in H295R, we performed a Western blot analysis (Figure 2B), showing an increase in the apoptosis-related proteins cleaved-PARP and phospho-HSP27 induced by mitotane treatment. Conversely, in the presence of NuSerum 2.5% or BSA 100 µM, the levels of these proteins were remarkably reduced. These results fit with our previous findings on the effects of NuSerum and BSA on mitotane action, thus confirming that BSA and a small amount of serum (principally composed of BSA) could remarkably reduce mitotane’s effect on H295R cells.

Most ACC cell models, such as SW13, MUC1, CU-ACC1, and ACC2, are considered mitotane resistant compared to H295R, and they are usually maintained in high serum/BSA conditions (FBS 5–10%) [18,19,20]. Our idea was that a “high-serum setting” could affect the estimation of mitotane activity in vitro. To test this hypothesis, we performed experiments on the SW13 cell line without serum, with NuSerum 2.5%, with FBS 10%, and with BSA 100 μM, as for the analysis of the H295R strain. In agreement with the results obtained in the H295R cell line, the addition of serum dramatically reduced the mitotane effect on the SW13 cell line (Figure 2C). The normal FBS 10% growth condition was able to block mitotane more than the other conditions, resulting in an IC50_24_ of 121.4 µM [95%CI: 85.43 to 172.4 µM] compared to 73.32 µM for NuSerum 2.5% [95%CI: 33.57 to 160.1 µM] and 10.83 µM for mitotane alone [95%CI: 8.579 to 13.66 µM]. A similar effect was also observed with BSA 100 µM.

### 2.2. Study of the Mitotane/BSA Interaction

**Figure 3:**
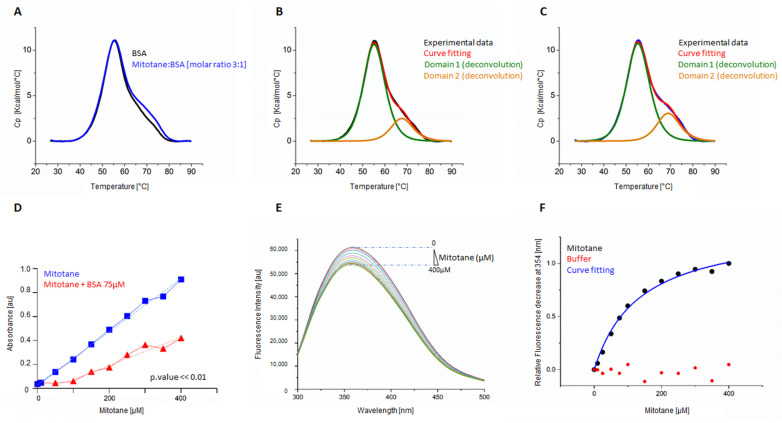
Mitotane/BSA interaction. (**A**) Calorimetric profiles of BSA in the absence (black line) and presence (blue line) of mitotane at 3:1 ligand/protein molar ratio. (**B**) Experimental line (black line), fitting (red line), and deconvolution of the BSA profile in the absence of ligand with two components according to a non-two state model. Domain 1 (green line), domain 2 (orange line). (**C**) Experimental curve (blue line), fitting (red line), and deconvolution of the BSA profile in the presence of ligand with two components according to a non-two state model. Domain 1 (green line), domain 2 (orange line). (**D**) Absorbance analysis of 96 wells with mitotane scale [0–400 μM] with or without BSA 75 μM. In light blue, the mitotane regression analysis and in light red, the corresponding regression analysis with 75 μM BSA calculated starting with mitotane 100 μM (regression analysis: dashed light blue line vs. dashed light red line; F = 341.3, DFn = 1, DFd = 64, *p* value << 0.01). (**E**) Fluorescence spectra of BSA solution (75 µM) in the presence of mitotane (T = 298 K, pH = 7.40, λex = 280 nm). The concentrations of mitotane were 0, 10, 25, 50, 75, 100, 150, 200, 250, 300, 350, and 400 μM. (**F**) Experimental points derived from plotting relative fluorescence quenching of BSA in the presence of mitotane (black points). Red points show the same additions performed with only buffer. The blue curve represents the fitting of the experimental points to determine the dissociation constant, Kd, of 134.2 ± 13.8µM. Absorbance and fluorescence are expressed in arbitrary units [au].

In order to assess whether mitotane binds to BSA, differential scanning calorimetry experiments (DSC) were carried out. Figure 3 shows the denaturation profile of BSA in the absence and presence of mitotane. The thermal profile for BSA shows a first peak at 55.2 °C and a shoulder at 67.7 °C (Figure 3A). The melting temperatures observed for the BSA peaks were in line with previous denaturation studies [21,22,23,24,25].

The thermograms obtained from the denaturation experiments can be deconvoluted into a sum of two contributions that represent the two domains of BSA that undergo separate non-two-state unfolding processes [26,27,28,29], which are indicative of the formation of intermediates state during the denaturation process (Figure 3B,C). The addition of mitotane in solution at a ligand/protein molar ratio of 3:1 determined no change in terms of Tm and unfolding enthalpy for the first domain. When the second domain was analyzed, while the Tm and the unfolding enthalpy were unchanged, both the Tm (68.04 ± 0.03 °C for BSA and 69.10 ± 0.02 °C for BSA with mitotane) and the ΔH were increased (entire thermogram: a difference of 4.5 × 10^4^ cal/Mol. For second domain: 2.98 × 10^4^ cal/mol ± 910 for BSA and 3.34 × 10^4^ cal/mol ± 925 for BSA with mitotane), suggesting the formation of a protein–ligand complex and confirming the hypothesis of binding of mitotane to BSA (Figure 3B,C). As further confirmation of a complex formation between the drug and the protein, a clear mitotane precipitation in the presence of 75 μM BSA was detected only at a concentration of mitotane > 100 μM, whereas the precipitate was already visible at 50 μM in the absence of BSA. This observation suggests a role of BSA in maintaining BSA in solution, as also confirmed quantitatively in the absorbance analysis (Figure 3D). Mitotane was poorly soluble in water (Figure 3D, blue line). The presence of 75 μM BSA in the solvent reduced the scattering measured at 490 nm, indicating an increase in the solubility up to a concentration of 100 μM (Figure 3D, red line). Furthermore, even when the effect due to BSA was saturated and mitotane began to precipitate, the linear relationship proceeded with a significantly smoother slope (Figure 3D, regression analysis: light blue line vs. light red line; F = 341.3, DFn = 1, DFd = 64, *p* value << 0.01).

Tryptophan (Trp) residues in the protein structure can be used to monitor changes in the microenvironment that are correlated with the binding of a molecule in solution. The fluorescence emission spectra of BSA in the presence of increasing concentrations of mitotane are shown in Figure 3E,F. The fluorescence spectra indicated that BSA had an intrinsic fluorescence band at 354 nm when excited at 280 nm (Figure 3E). The addition of mitotane at concentrations of 10–400 µM induced the quenching of the fluorescence intensity of BSA and caused a slight blue shift (1 nm, Figure 3E). The change in fluorescence corresponded to a change in the environment surrounding Trp residues due to the binding of mitotane and the energy transfer. Furthermore, when the BSA fluorescence decrease was plotted as a function of increasing mitotane concentration, a clear binding curve, totally absent in the control reaction (titration of buffer into BSA), was observed (Figure 3F). The fitting of the binding curve resulted in a dissociation constant Kd of 134.2 ± 13.8 µM.

### 2.3. Docking Experiments

**Figure 4:**
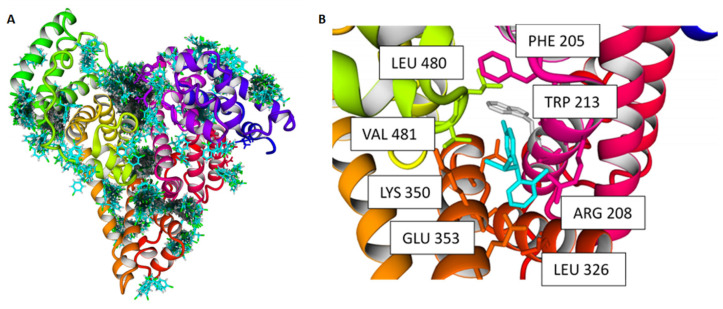
Docking of mitotane to BSA. (**A**) The crystal structures of BSA PDB ID: 3V03 was docked using the VINA algorithm, embedded in YASARA. Docking analysis revealed that mitotane binds both within the internal grooves of BSA and subsequently to the surface of the structure. (**B**) Best pose of mitotane binding to BSA. This represents the best complex obtained after 999 docking runs. Mitotane is represented in cyan. The other residues participating in the interaction with mitotane are also labeled.

We then performed further in silico docking to analyze the ability of mitotane to interact with the crystal structure of BSA. In order to perform an unbiased analysis of mitotane, a cubic simulation cell was built on the crystal structure of BSA and extended by 5.0 A on all sides. The docking results from 999 runs using the YASARA embedded VINA algorithm [30,31] produced all favorable poses, indicating a promiscuous interaction between BSA and mitotane. When the poses were clustered, 123 distinct complex conformations were found, differing by at least a 5.0 A heavy atom RMSD after superposing on the receptor (Figure 4A). Among all the poses, the best binding energy was found to be 7.21 kcal/mol (Figure 4B). This pose showed how Trp 213 was influenced directly by the binding of mitotane (Figure 4B). Together, the in silico docking simulations were therefore consistent with the fluorescence analysis and supported the hypothesis that BSA is a carrier of mitotane.

### 2.4. Study of the Influence of Human Serum on the Pharmacological Effect of Mitotane

**Figure 5:**
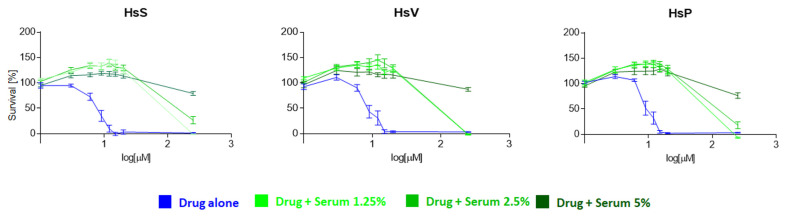
Effect of addition of human serum on mitotane activity in H295R cell model. Concentration–response curve of drug-induced inhibition of cell viability. H295R cells were cultured for 24 h with a range of mitotane concentrations (0–250 µM). This standard condition for each drug was “perturbed” by adding a fixed amount of different concentrations of human serum (1.25, 2.5, and 5%) utilizing three different serum sources: commercial human serum (HsS), a healthy volunteer (HsV), and a patient with ACC (HsP). Technical quadruplicates were performed for each drug condition and the experiments were replicated at least 2 times.

Human plasma has a high content of proteins and albumin represents half of the total protein content. In the bloodstream, the main functions of albumin are to regulate plasma oncotic pressure and to transport endogenous/exogenous proteins and molecules, including drugs. For this reason, we tested whether human serum could affect the in vitro mitotane efficacy in H295R cells. Three different serum sources were used at different concentrations (0%, 1.25%, 2%, and 5%): (A) commercial human serum (HsS) HIV tested from multiple blood types and multiple genders (S4200 BioWest), (B) serum from a healthy volunteer (HsV), and (C) serum from a patient with ACC (HsP). In agreement with what we have observed with the addition of NuSerum/FBS and BSA, the analysis of cell viability showed that all three human serum sources affected mitotane’s action even at the lowest concentration, blocking the normal sigmoid graph shape (Figure 5). The effect was similar to the on–off behavior observed in previous experiments, although even 5% human serum was able to partially block the maximal dose of mitotane used as a death control. No difference was observed when replacing serum with plasma, which also contains coagulation proteins.

**Figure 6:**
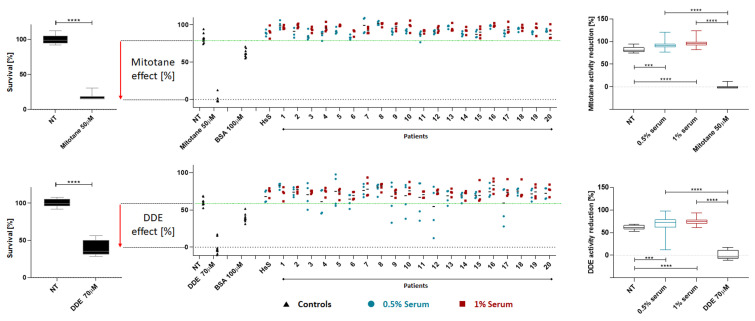
Effect of patient sera on mitotane/DDE activity in H295R cell line. Twenty sera derived from untreated patients with ACC were used to evaluate serum interference on mitotane/DDE activity. Approximately 50,000 cells per well were seeded in a 96-well plate in DMEM-F12 complemented with ITS but in the absence of serum. After 24 h, the cells were spiked with drug (50 µM mitotane or 70 µM DDE) and 0.5% or 1% of serum derived from each different patient. Controls in each plate were: no serum/untreated cells (NT); no serum/drug alone; drug and 0.5% or 1% human standardized serum (HsS); drug and BSA 100 µM. Technical duplicates were performed for each condition and the experiments were replicated at least 2 times. “−” indicates the mean value calculated for the 4 measures of each patient. *** indicates a *p* value < 0.01; **** indicates a *p* value << 0.01.

To assess if this result was patient-specific or a general effect of serum, we performed the experiments on mitotane/DDE activity by evaluating 20 sera derived from untreated patients with ACC. In the absence of serum, mitotane 50 μM treatment for 24 h reduced cell vitality to 16%, while the addition of BSA 100 μM was able to block 64% of the drug activity, restoring 80% of cell viability (Figure 6, left upper panel). We observed a similar but less intense effect in the experiments with DDE 70 μM for 24 h, which reduced cell vitality to 34%. In this case, the addition of BSA 100 μM was able to block 44% of DDE activity, maintaining 78% of cell vitality (Figure 6, left lower panel). All the human sera were able to completely and significantly block the drug effect at 1% concentration (both Welch’s *t* test *p* value << 0.01). Mitotane was more sensitive to the presence of human serum, as we obtained the same blocking effect with only a 0.5% serum concentration (*p* value < 0.01); in contrast, we observed more variable results at this concentration for DDE (*p* value < 0.01; Figure 6, middle/right panels). Interestingly, our analysis of cell populations treated with patient sera revealed a significantly higher survival rate in comparison to serum-free/untreated (NT) cells. This increase in cell viability can be attributed to the presence of several growth-inducing factors in patient sera that reduce cellular stress due to starvation (all contrasts evaluated are shown in the right panels of Figure 6; all *p*-values from Welch’s *t* test <0.01).

## 3. Discussion

The diverging results derived from in vitro experiments on mitotane activity in identical cell lines highlight the need for a standard methodology to perform these analyses. In this study, we thoroughly investigated how the pharmacological effect of mitotane and its metabolites is influenced by the presence of “confounding factors”. Our experiments clearly indicated that both albumin and human serum are able to suppress the pharmacological effect of mitotane in vitro. This suppression led to a shift from the typical sigmoidal dose–response curve to an on–off response, where the maximal drug effect was only observed at oversaturated concentrations. This blocking effect was independent of the methodology used to evaluate the drug’s toxic effect. Furthermore, the greater the effect of the drug, the better the inhibition of its toxic activity was observed. These data confirmed and largely expanded the results obtained by Hescot et al., who also identified a similar inhibitory effect [14].

Notably, albumin and lipoproteins are the most abundant proteins in culture serum and could also be over-represented in human serum. Albumin can undergo various post-translational modifications that can alter its physical characteristics, e.g., several liver and kidney diseases have been related to its oxidation state [32]. Albumin has an important role in maintaining oncotic pressure and acts as a transporter for many substances, such as calcium and some drugs. In addition, its ability to promote various organic reactions and to bind different reactive species supports the definition of albumin as a promiscuous biocatalyst [33]. In the present study, we showed a mitotane–albumin interaction that affects the calorimetric characteristics of BSA while simultaneously increasing the water solubility of mitotane. Furthermore, fluorescence quenching experiments show the binding of mitotane to BSA in solution with a Kd of 134.2 µM, and docking analysis confirms that mitotane can efficiently bind the surrounding Trp 213, but it can also interact more weakly with the rest of the protein. In our experiments, this interaction was also observable in water alone, which, compared to a biological fluid, lacks cofactors that could contribute to and/or favor the binding. Interestingly, the BSA–mitotane interaction alters the biological effect of the drug. In our experiments, the addition of 100 µM BSA to cells treated with mitotane or DDE was sufficient to block most of the drug activity. This protein concentration was significantly lower than that present in human plasma, which typically ranges around 0.5–0.8 mM. Physiologically, albumin is constantly exposed to several stresses that can alter its physical characteristics [32], but little is known about how drugs, such as mitotane and derivatives, could interact with or modify albumin [33]. Our data highlighted how a deeper understanding of this interaction could be useful in identifying drug resistance and developing biomarkers for predicting patients’ responses to drugs. For this reason, as also suggested by Weaving et al., the standardization of the inter-laboratory quantification of plasma albumin will likely be essential in the future [34].

In our experiments, serum was able to block mitotane’s action, regardless of the extraction source (commercial multigender mix, healthy donors, or patients with ACC). Interestingly, the effect of serum derived from a healthy donor or ACC patient was more effective than albumin alone. This could be explained considering that, although albumin may be responsible for much of the mitotane-blocking effect, serum is a complex mixture. Its composition may depend on physiological activities, such as hormonal activity of the assessment of nutritional status, or on pathological conditions, such as cancer or liver disease. This multitude of substances could probably interact with mitotane and its metabolites, reducing the active drug necessary to counteract the ACC.

Our data highlighted that the outcome of in vitro experiments is strongly affected by confounding factors that could hamper the correct assessment of molecular mechanisms. The present findings demonstrate that if the SW13 cells, the archetype of mitotane-resistant cell lines, were cultured in the same serum/media concentration as the H295R cells, the archetype of mitotane-sensible cell lines, a comparable mitotane IC50_24_ would be obtained. These data indicate that the condition of “mitotane resistance” observed in some cell lines could result from specific experimental conditions, thus inducing a bias that could hamper the correct assessment of the actual mitotane effect. These findings confirm and extend the data from previous studies showing that culture conditions, such as the cell culture medium, different serum brands, and/or additive concentrations, strongly influenced mitotane’s pharmacological effect [15,16,17].

## 4. Materials and Methods

Drugs and chemicals. Mitotane was dissolved in ethanol absolute in a stock solution of 156 mM and stored at −20 °C. DDA and DDE were dissolved in DMSO in a stock solution of 132 and 157 mM, respectively, and stored at −20 °C. All drugs were provided by Selleckchem Chemicals.

Cell lines and culture conditions. H295R and SW13 cells were kindly provided by Prof. S. Sigala (Department of Molecular and Translational Medicine, University of Brescia, Brescia, Italy) and cultured at 37 °C with 5% CO_2_ according to the American Type Culture Collection (ATCC) instructions. Media and supplements were purchased from Euroclone. Specifically, H295R was grown in DMEM: F12 50:50 medium (Gibco: Thermo Fisher Scientific, Waltham, MA, USA) supplemented with 2.5% NuSerum (Corning #355100, Hongkong, China), penicillin/streptomycin (Gibco: Thermo Fisher Scientific), and ITS Premix (Corning #354350). Of note, we used ITS instead of ITS+, because this last contains bovine serum albumin (BSA) which was one of the additive substances evaluated in our experimental conditions. There are no differences in the growth rate of cell lines maintained in a medium containing ITS or ITS+. SW13 cells were grown in DMEM medium supplemented with 10% fetal bovine serum (FBS) and penicillin/streptomycin (Gibco: Thermo Fisher Scientific). Unless indicated, all experiments were conducted in the absence of serum. Cell lines were periodically tested for mycoplasmas and authenticated with genetic profiling using polymorphic short tandem repeat loci with the PowerPlex^®^ 16 System (Promega, Madison, WI, USA) and Applied Biosystems 3130 genetic analyzer (Thermofisher, Waltham, MA, USA).

Cell viability and apoptosis analysis. The 3-(4,5-dimethylthiazol-2-yl)-5-(3-carboxymethoxyphenyl)-2-(4-sulfophenyl)22H tetrazolium (MTS) viability assay was performed as follows: cells were cultured for 24 h testing different drug concentrations (mitotane and DDE 0–250 µM; DDA 0–1 mM); for each drug, the range of increasing concentrations was selected to maximize the sigmoidal pattern of the dose–response graph. For each drug, this standard condition was “perturbed” by adding a fixed amount of an additive substance to each curve concentration point (e.g., BSA 100 µM, NuSerum 2.5–5%, human standardized serum [HsS, Human Serum HIV tested, #S4200; Biowest, Nuaillé, France], and patient or healthy donor serum). MTS was added after drug incubation, according to the manufacturer’s instructions (Promega), and the absorbance at 490 nm was recorded with an iMark™ Microplate Absorbance Reader (Biorad, Hercules, CA, USA). Technical quadruplicates were performed for each drug condition and the experiments were replicated at least 2 times. The half maximal inhibitory concentration at 24 h (IC50_24_) is a measure of the effectiveness of a molecule/drug inhibiting a specific biological or biochemical function. In our analysis, this is the concentration of a single drug (mitotane, DDE, or DDA) or combinations of a drug and an “additive substance” endowing a viability reduction of 50% after 24 h of drug exposure. IC50_24_ values were calculated by evaluating the viability data with a sigmoidal dose–response analysis using GraphPad software 7. Since IC50_24_ was generated using an algorithm that depends on the starting cell number and other experimental conditions, we maintained the drug-alone condition for every replicate in each experiment.

To measure apoptosis, H295R cells (1.5 × 10^5^ cells/well) were seeded in 24-well plates in complete medium; after 24 h of incubation with the appropriate concentration of each drug in the study and with or without additive substances, the cells were detached and decorated with FITC-Annexin V (Invitrogen, Waltham, MA, USA) antibody. FACS analysis was performed with a cell sorter/FACS facility (Becton Dickinson, Hongkong, China).

Western blotting analysis. At 24 h after treatment, cells were lysed with Laemmli modified buffer (2.5% SDS, 125 mM Tris-HCl pH 6.8) previously heated to 95 °C. The protein concentration was determined using the QPRO BCA protein assay kit (Cyanagen, Bologna, Italy). An equal amount of lysate was resolved on a 10% acrylamide gel and transferred onto a PVDF membrane (Biorad, Hercules, CA, USA). Filters were incubated overnight at 4 °C with 1:1000 primary antibodies: cleaved PARP (Cell Signaling Technology (CST), #5625, Danvers, MA, USA); phospho-HSP27 (CST, #2405); β-actin (Sigma-Aldrich, A5316, St. Louis, MO, USA). Anti-rabbit IgG/HRP (CST, #7074) and anti-mouse IgG/HRP (CST, #7076) were used as secondary antibodies (1:8000). Enhanced chemiluminescence method (Clarity ECL, Biorad, Hercules, CA, USA) was used for protein bound detection.

Calorimetry. Differential scanning calorimetry (DSC) experiments were carried out using a Microcal VP-DSC instrument from Malvern Panalytical Ltd. (Malvern, UK) with the following set-up: 25–90 °C temperature gradient, 90 °C/h scan rate, 10 min pre-scan equilibration at 25 °C [35,36,37]. A solution of 75 µM BSA was prepared in 10 mM KPi pH 7.4. The unfolding temperature Tm and the ΔH were determined by fitting the data with the OriginPro embedded program suite (OriginLab Corporation, Northampton, MA, USA). Cooling and reheating of the samples were performed to obtain the background for buffer subtraction. Replicate runs did not vary more than 0.15 °C. DSC analysis in the presence of mitotane was conducted using 75 µM BSA and 225 µM mitotane, which was found to be the solubility limit for mitotane in buffer solution.

Absorbance analysis. Precipitation of mitotane in aqueous solution was tested in a 96-well ELISA plate in quadruplicate for each concentration of mitotane [0–400 µM] with or without 75 µM BSA. The absorbance was evaluated using a 490 nM wavelength with the iMARK instrument (Biorad, Hercules, CA, USA). To avoid misinterpretation due to mitotane deposition at the bottom of the wells, the plate was shaken for 5 s before absorbance recording. Changing the wavelength did not affect the observed results.

Fluorescence Quenching Assay. Mitotane was tested for its ability to bind to BSA using fluorescence quenching. In this assay, mitotane quenches the protein’s intrinsic fluorescence produced by the tryptophan fluorophores. Measurements were performed in a quartz cuvette using a fluorescence spectrometer LS55 (PerkinElmer, Waltham, MA, USA). Fluorescence emission spectra were monitored in the 300–500 nm range using a scan rate of 200 nm/min upon excitation at 280 nm. The excitation and emission slits were arranged to 8.0 and 10.0 nm, respectively. The protein was dissolved in water at a concentration of 75 μM. Samples were measured in the absence and presence of mitotane at 25 °C.

Docking Experiments. Ligand and protein structure (PDB ID: 3V03 [38]) were prepared using the YASARA structure package [39]. The mitotane structure was then optimized through energy minimization, as previously reported [40]. The AMBER 03 force field was employed throughout all simulations. In the docking experiment, the substrate is originally outside the simulation cell and is placed inside the cell by exploiting the VINA algorithm, resulting in a series of binding modes classified by the binding energy outputs [40]. Among these binding modes, the complex protein–ligand bearing the highest binding energy, calculated with YASARA as the mechanical energy required for disassembling a whole into separate parts (where positive energies indicate stronger binding and negative energies equate to no binding), was selected and further refined with local docking. After docking, the results were sorted by binding energy.

Human sera. For the experiments, we selected 20 ACC patients and one healthy individual from a large biorepository of biological samples available at the University of Turin, Internal Medicine—San Luigi Hospital (Orbassano, Turin, Italy). Blood samples were drawn in Vacutainer tubes (BD #368968) and, after a minimum clotting time of 30 min, centrifuged for 15 min at 1300 g. Serum was anonymized and immediately frozen at –80°. Written informed consent, approved by the local ethics committee, was obtained from each enrolled subject. Twenty sera derived from untreated ACC patients were used to evaluate their interference with mitotane or DDE treatment. We seeded approximately 50,000 cells per well in a 96-well plate in DMEM-F12 complemented with ITS but in the absence of serum. After 24 h, the cells were spiked with drugs (mitotane 50 µM or DDE 70 µM) and 0.5% or 1% of each patient’s serum. As controls, in each plate were also present: no serum and untreated cells (NT), drug and 0.5% or 1% HsS, or drug and BSA 100 µM. Technical duplicates were performed for each condition and the experiments were repeated at least 2 times.

Statistical Analysis. The associations between the treated cell populations and the respective controls were evaluated using the Welch’s *t* test. All associations with a *p* value < 0.05 were considered statistically significative. Statistical analysis was performed using R statistical software version 4.3.0 (R Foundation for Statistical Computing, http://www.r-project.org/foundation/, accessed on 21 November 2023) [41]. “<<” mathematical symbols are used to indicate that the *p* value was “much less than”.

## 5. Conclusions

In conclusion, we have for the first time demonstrated that albumin does not only act as an inert drug carrier when mitotane or its metabolites are present. Indeed, the biological activity of these drugs was strongly inhibited by the drug–albumin interaction observed. This blocking effect was independent of the methodology used to evaluate the toxic effect of the drug and altered the evaluation of the drug sensitivity of the cell lines. For these reasons, our results support the concept that the measurement of free mitotane may be a more reliable marker of the activity of the drug in clinical practice. Furthermore, the identification of new drugs, less unstable than mitotane and its derivatives, is an unmet clinical need to obtain a more effective treatment for ACC. The development of an intelligent method to deliver mitotane that increases the free active form of the drug may be an appealing alternative, as also suggested by M.S. Haider et al. [42]. In the future, also starting from the experimental results obtained in vitro and through a personalized medicine approach, we will have to decipher the specific molecular fingerprint of the patient by identifying who will positively respond to the ACC therapy.

## Data Availability

Data is contained within the article.
